# Barriers and sources of information in prostate cancer screening at a South African hospital

**DOI:** 10.4102/jphia.v16i1.666

**Published:** 2025-04-12

**Authors:** Boitumelo M. Komane, Annah Mosalo

**Affiliations:** 1 Department of Health Studies, College of Human Sciences, University of South Africa, Pretoria, South Africa

**Keywords:** barriers, cancer, experiences, men, prostate screening, pain control

## Abstract

**Background:**

Prostate cancer is a worldwide problem affecting men globally, particularly in low- and middle-income countries. However, research pertaining to barriers to screening uptake and sources of information regarding prostate cancer is limited in South Africa.

**Aim:**

The objective of the study was to explore barriers to screening and sources of information among men attending urologic clinic at a specific tertiary hospital in Gauteng.

**Setting:**

Urology clinic at a specific tertiary hospital in Gauteng.

**Methods:**

A qualitative, exploratory study was conducted with 19 conveniently selected men attending the specific clinic. Data were analysed using thematic analysis.

**Results:**

Three themes emerged which were (1) barriers to screening, (2) sources of information regarding prostate cancer and (3) symptoms experienced during screening.

**Conclusion:**

The study provided evidence regarding the lack of privacy, pain control, fear of the procedure and embarrassment as being barriers towards screening. Failure of healthcare practitioners to recommend screening when in contact with patients eligible for screening also contributed as a barrier. Media and pamphlets played a role as sources of information regarding awareness pertaining to the disease.

**Contribution:**

The study findings have a potential to help healthcare practitioners to be proactive and recommend screening for men who are eligible or might be at risk. The findings will also help healthcare practitioners to be sensitive and exercise caution in maintaining privacy during screening, and that men are informed regarding what screening procedures entail to gain their cooperation.

## Introduction

Prostate cancer is one of the major cancers affecting men globally, with high prevalence, mortality and healthcare costs.^1,2^ Its impact is increasingly felt and it has serious effects within communities and on healthcare systems worldwide.^3^ The burden of prostate cancer remains substantial across the globe as well as in Africa, and South Africa.^4^ Studies reported the burden of prostate cancer to be high among black men compared to other ethnic populations.^5,6,7^ This underscores the need for proactive measures for the early detection and management of prostate cancer.

In response to the increased burden of prostate cancer, screening programmes are available to mitigate the severity of the disease.^7^ The most commonly utilised screening tools include digital rectal examinations and prostate-specific antigen (PSA) tests.

The purpose of the screening is to identify prostate cancer at an early stage when options of treatment would be highly effective.^7^ Although screening is beneficial, it is important to understand the barriers to screening and sources of information to optimise the screening programmes. However, there is little research on the barriers to screening uptake and sources of information regarding prostate cancer at the specific urologic clinic in Gauteng.

According to Benedict et al.,^8^ available studies in Africa reported that the decision of patients to undergo prostate cancer screening is influenced by various factors such as individual beliefs, healthcare practices and societal norms. Additionally, for some patients, the decision to screen for prostate cancer may result from the symptoms experienced such as erectile dysfunction and urinary incontinence. Other factors that influence patients’ decisions to screen for prostate cancer, according to African studies, include referrals by healthcare practitioners and experience of a family member or relative who had prostate cancer.^9^ Furthermore, the perceptions of men towards screening were reported as a significant influence for men’s participation and the likelihood of engaging in screening.^9^ Hence it is important to explore the barriers to screening and sources of information regarding screening uptake among men undergoing prostate cancer screening to unravel the challenges related to screening uptake and participation.

The researchers in the study were not sure what the barriers to screening and sources of information regarding prostate screening in South Africa were, particularly at the specific tertiary hospital chosen for this study. Hence, the decision to conduct a study to explore the barriers to screening and sources of information with regard to prostate cancer at a specific hospital in Gauteng, South Africa. Gauteng, as the most populous province in South Africa, provides a unique setting with diverse cultural norms, demographics and healthcare infrastructure. This has the potential to inform policy development and targeted intervention to improve screening uptake in the region.

## Research methods and design

### Study aim

The study aims to explore the barriers and sources of information towards prostate cancer screening among men at the urologic clinic at the specific academic hospital in Gauteng.

### Study design

A qualitative exploratory study was conducted to explore barriers to prostate cancer screening and access to sources of information regarding the disease at a specific hospital in Gauteng province. Qualitative research was suitable as it focuses on the understanding and in-depth inquiry of the phenomenon being studied. Qualitative research was suitable as researchers could not trace studies within their context exploring barriers and sources of information. Face-to-face in-depth interviews were conducted to explore the barriers to screening and sources of information among men attending prostate cancer screening at the urologic clinic at the specific tertiary hospital.

### Setting

The study was conducted at a urology clinic at a specific academic hospital located in Soweto township, an urban area in Gauteng province, originally established as a segregated settlement for black South Africans during apartheid. This hospital is one of the largest in South Africa and is renowned for its specialised services, including a dedicated urology department. It receives referrals from secondary care hospitals, and primary healthcare across Gauteng province. The urology clinic offers a range of services, such as bladder care, dialysis and treatment for penile and prostate cancer, among other urological conditions affecting men. Patients are typically referred from nearby secondary care facilities, while some come as walk-ins from the local Soweto area and neighbouring rural settlements. The majority of the clinic’s patients are from middle- and low-income communities who lack medical insurance for private hospital care.

### Population

The study population consisted of all men 40 years and older who attended the urology clinic at the specific academic hospital between February 2022 and July 2022.

### Sampling

Purposive sampling technique was adopted to select men who met the study criteria. Participants were present at the hospital for prostate cancer screening and were easy to reach and invited to participate in the study. The sample size was determined by data saturation.^10^ The researcher aimed to interview 15 participants, allowing for contingencies in case of those who were unable to complete the study because of factors like excessive pain or being very ill to participate or a decision to withdraw. The final sample consisted of 19 men including two who were part of the pre-test as there were no changes to the interview guide, as well as two last interviews to ensure that data saturation was reached, aged 40 years or older who underwent prostate cancer screening at the specific tertiary hospital between February 2022 and July 2022, at which point data saturation was achieved.

### Inclusion criteria

The study included men who met the following criteria: they were 40 years or older, underwent prostate cancer screening at the specific tertiary hospital between February 2022 and July 2022 and were referred to the hospital for this purpose. Additional inclusion criteria were having a family history of prostate cancer, the ability to provide informed consent, a positive PSA test result and being in the workup stage without a confirmed diagnosis of prostate cancer.

### Exclusion criteria

Men with the following characteristics were excluded from the study: those under 40 years attending for other urological conditions, men already undergoing prostate cancer treatment, those unable to provide informed consent, men over 40 years who were too ill to participate and those presenting with psycho-emotional issues.

### Data collection

Data collection occurred during the COVID-19 pandemic. All protocol recommended by the World Health Organization (WHO)^11^ were followed. These measures included hand hygiene, wearing clean face masks, physical distancing, environmental cleaning and the management of COVID-19 patients (WHO 2019). Since the hospital was already adhering to these protocols, the researcher ensured they were rigorously followed for the purpose of the study.

The interview guide was validated by the staff at the urology clinic, including urologists, nurses involved in day- to-day patient care and the study supervisor, who is trained in oncology. The guide was pre-tested with two patients to assess its clarity, and no additional recommendations were made. The data from the pre-tests were included in the overall dataset. Data collection occurred during February 2022 to July 2022. Prostate cancer screening at the urology clinic was conducted only on Wednesdays, so the researcher opted to interview two patients per week on these days. The researcher sourced the support of the clinic staff to invite men attending the urology clinic to participate in the study. Informed consent, including permission to record the interviews, was obtained prior to each interview.

Individual interviews were conducted in a private room arranged to ensure privacy and confidentiality, aiming to prevent any stigma associated with the patients’ illness. Each interview lasted approximately 30 min – 40 min. The interviews were conducted in isiZulu and Setswana, the common languages spoken by the local community. The primary researcher, who resides in a similar community and is fluent in both isiZulu and Setswana, conducted all the interviews, including those in English for participants who did not speak the indigenous languages.

### Data analysis

The researchers transcribed all interviews verbatim. To ensure accuracy, a second researcher reviewed the first two transcripts, comparing them with the original transcriptions to confirm that no meaning was lost and that all relevant codes were captured for theme development. Additionally, an independent intercoder, who was not involved in the study but was familiar with research and fluent in the languages used, coded several transcripts. This step was taken for validation and to ensure consistent meaning across the data.

Data analysis was conducted manually by the researchers and followed an iterative process. A thematic analysis was performed to address the study objectives, guided by a codebook developed to identify codes and categories for theme development. A meeting was then held between the intercoder and the researchers to discuss the findings and reach a consensus. To maintain confidentiality, the intercoder signed a confidentiality agreement, ensuring that no information was disclosed outside the study.

### Trustworthiness

The principles of credibility, dependability, conformability and transferability were used to assess the trustworthiness of the study.^12^ Credibility was ensured by conducting multiple visits at the urology clinic of the specific academic hospital and collecting data until saturation was reached. The multiple visits were to acquaint the researcher with the processes in the clinic including familiarisation with the patients who are attending the clinic. Also, prolonged engagement between the interviewer and participants facilitated a deeper understanding and building of trust. Transferability was ensured by providing a thick description of the study to enable the reader to evaluate whether the study can be replicated to their contexts. The quality of the data was maintained through field notes, effective communication, the use of good quality recording equipment, and a comfortable environment to ensure dependability. Conformability was ensured by prolonged engagement in the field with participants at the specific academic hospital; this allowed the researchers to build rapport with participants, which in turn facilitated obtaining rich, useful, and accurate data.

### Ethical considerations

Ethical clearance to conduct this study was obtained from the University of South Africa College of Human Sciences Research Ethics Review Committee (No. 61263079_CREC_CHS_2021).

Data collection began after receiving ethical clearance from the College Research Ethics Committee and permission from Department of Health (DOH) in order for the head of the urology department to grant permission to conduct the study. Permission at the study site was granted by the specific academic hospital. Participants in the study signed informed consent forms indicating their willingness to participate and they consented to the recording of the interviews.

The ethical principles of informed consent, anonymity, privacy, confidentiality, non-maleficence, beneficence and justice were maintained. The principle of beneficence was upheld by ensuring that, although participants did not receive direct benefits, the findings would be used to increase prostate cancer screening uptake through dissemination to public health officials and policymakers. The principle of respect for persons was honoured by respecting participants’ decisions, including their right to withdraw from the study at any time without consequence. The principle of non-maleficence was maintained, as no harm resulted from participation. A psychologist was arranged in advance for counselling to be available should there be a need during participation in the study. However, this was never utilised. Justice was ensured by adhering to legal standards and enabling participants to ask questions and make informed decisions about their involvement while continuing to access healthcare benefits. Only the researchers of the study including the co-coder could access the raw data of this study. The co-coder signed a confidentiality binding agreement form agreeing not to share the data with third parties. The data for this study are stored on University OneDrive and a password-protected computer.

## Results

A total of 19 participants between the ages of 54 years and 84 years participated in the study (Table 1). Six of the participants were Zulu, followed by four South Sotho, two Swazi and two Tsonga. There was one participant from each of mixed race group, Ndebele, North Sotho, Xhosa and another unspecified group. Most of the participants (*n* = 15) came from urban regions, while four came from rural areas and were referred to the hospital. Fourteen of study participants were married, while two were culturally married, one cohabiting, one in a stable relationship and one being single. Ten of the participants completed their tertiary education, while seven participants completed Grade 11–12 and only two men completed Grade 8–10. Fifteen of the participants were on state pensioners grant and received a monthly pension income of R2000.00 or more. The other four participants were unemployed; however, of these, two were receiving a monthly income of R2000.00 or more, while the other two did not have any monthly income.

**TABLE 1:** Demographic characteristics of the participants (*N* = 19).

	*n*	%
**Cultural group**		
Mixed race people	1	5.26
Ndebele people	1	5.26
North Sotho people	1	5.26
South Sotho people	4	21.05
Swazi people	2	10.53
Tsonga people	2	10.53
Xhosa people	1	5.26
Zulu people	6	31.58
Other	1	5.26
**Region**		
Rural	4	21.05
Urban	15	78.95
**Age (years)**		
50–59	4	21.1
60–69	7	36.8
70–79	7	36.8
80+	1	5.3
**Marital status**		
Married	14	73.68
Co-habiting	1	5.26
Culturally married	2	10.53
Stable relationship	1	5.26
Single	1	5.26
**Number of children**		
Average number of children	3	-
**Educational level of participant**		
Grade 8–10	2	10.53
Grade 11–12	7	36.84
Tertiary	10	52.63
**Employment status of participant**		
Pensioners	15	78.95
Unemployed	4	21.05
**Monthly income of participant**		
None	1	5.26
R1.00–R2000.00	17	89.47
R2001.00–R2500.00	1	5.26

### Presentation of findings

Three themes emerged from the data (Table 2), namely, (1) barriers to screening for prostate cancer, (2) sources of information regarding screening and (3) symptoms experienced during screening. Once themes had been generated from the data, several categories were developed from participants’ quotes and they presented similar issues.Mixed raceThe quotes reflect the perspective of the participants at the specific tertiary hospital.

**TABLE 2:** Themes and categories of the study findings.

Themes	Categories
1.Barriers to screening	1.1. Fear, shyness and embarrassment 1.2. Lack of information regarding the disease
2.Sources of information regarding the disease	2.1. Media 2.2. Pamphlets
3.Symptoms experienced during screening	3.1. Pain 3.2. Discomfort

### Theme 1: Barriers to screening for prostate cancer

Participants in the study were asked to describe their barriers to screening of prostate cancer. These barriers to screening for prostate cancer emerged as a theme in the study. Categories that emerged were (1) fear, shyness and embarrassment, and (2) a lack of information regarding the disease. The following are excerpts as shared by the participants.

#### Category 1.1: Fear, shyness and embarrassment

Fear and embarrassment have been reported in this study as preventing men from undergoing screening. Participants reported that fear, shyness and embarrassment hindered them from screening for prostate cancer. Their responses were as follows:

‘I sometimes feel like maybe men know about this thing [*meaning screening procedure*] and what it is going to be done to them. Sometimes you find that I am a man my age ne! [*emphasising*] I am scared [*implying to be embarrassed*] to be inserted those fingers or sometimes.’ (Participant 13, 58 years, Unemployed)

‘Maybe they are scared! [*Implying being hesitant*] so they don’t come to check their prostate.’ (Participant 1, 66 years, Pensioner)

However, men in general tend to be private as they might be afraid or embarrassed. This is what the participants had to say:

‘They are shy! Yea! they are shy … This is how I see it. Some it is because they are shy! like *hai* [*exclaiming*], people will say I have prostate cancer you see.’ (Participant 3, 84 years, Pensioner)

‘Well men if you check they are embarrassed when it comes to these things. They have this thing that this is my life, this is my privacy, then to be checked by other people I will never be a man enough [*implying loss of dignity*], well I am going to be disrespected. Do you get me [*emphasising*]. so that is why sometimes some of them refuse.’ (Participant 5, 70 years, Pensioner)

‘You find that another man is embarrassed that at his age he will have to undress for a younger child [*implying young doctors*] [*the participant appeared truly embarrassed as he reported*].’ (Participant 7, 54 years, Unemployed)

#### Category 1.2: Lack of information regarding the disease

The study found that the lack of information has been identified as a barrier to screening. The participants in the current study also revealed that the lack of information about prostate cancer screening was a barrier to screening for the disease, as evidenced by the narratives shared:

‘Most of the time it is lack of knowledge you get my point! It is lack of knowledge! [*emphasising*] With me I just discovered that, I am 68 years now, I think I was 63 or 64, when I Googled. If I knew before then at the age of 40, I should have start checking! I would then tell those younger that at the age of 40 you should test.’ (Participant 18, 68 years, Pensioner)

‘I think you know what, I don’t know how I can put it … an un-educational behaviour [*implying people with low level education*]. I mean some people just want to act ignorant! You see if I say ignorant it’s that they know that it [*prostate cancer*] [*assuming they heard about it*] is there, but they want to put themselves on a side that ah! I will see it when it comes it comes on stage 4.’ (Participant 2, 57 years, Unemployed)

Others had this to say:

‘This should start from the community. Because the community! if you tell the community what prostate cancer is, how it happens then people will be able to know and go to the hospital and start to test prostate cancer early before it is too late!! [*emphasising*]. Yea!! it should start from the community not here at the hospital. Because if it starts from here, it means you are suffering already [*implying by then definite cancer has been diagnosed*].’ (Participant 13, 58 years, Unemployed)

‘So, we [*patients*] also like to go see traditional healers to make us some medications [*herbal concoctions or traditional medicine*] what is the use of going to the hospital because the traditional healer is like a jack of all traits [*meaning are able to cure*], and only to find out that the person knows nothing about this particular disease that you have and he cannot even understand!’ (Participant 16, 66 years, Pensioner)

‘Us black people, I am sorry to talk like this [*apologetic*], but we have our own beliefs, we don’t believe in these things. But honestly not knowing what you trust because you have to go to find help. For me there is nothing that I am scared of when I go to the doctor, I know the doctor will help me [*meaning that he trusts the doctors*]. It could be that I have wrong knowledge about something [*laughing*] appears embarrassed.’ (Participant 15, 62 years, Pensioner)

### Theme 2: Sources of information regarding screening

The second theme identified in the study was sources of information regarding prostate cancer. The information sources were (1) media, and (2) pamphlets from the health facility.

#### Category 2.1: Media

The media played a major role in communicating health issues in the study as most participants are exposed to the media. Accordingly, media were identified as sources of information regarding prostate cancer among some study participants. The following information was shared by the participants:

‘I have seen this on media as well, internet as well because I have been perusing it to even understand what I am going through, so I have been doing my own research as well.’ (Participant 17, 54 years, Unemployed)

‘From the radio and newspapers! [*emphasising*] they always say this, I am a person who hear [*listen to*] the news a lot, I also read a lot, so I sometimes come across these things.’ (Participant 16, 66 years, Pensioner)

‘My understanding due to the research I conducted on Google about prostate, I know that prostate cancer usually affects black men.’ (Participant 12, 76 years, Pensioner)

#### Category 2.2: Pamphlets

The participants reported that the health facility distributed pamphlets as a way of conveying knowledge or communicating health information to patients. One participant reported that they received a pamphlet from the local clinic, while others indicated that the pamphlet was provided at the specific academic hospital. Their narratives were as follows:

‘At the clinic most of the time they encourage men that they must go for prostate screening, especially in the townships they have notes, [*implying pamphlets*], there are pamphlets, and they encourage men that at the age of 40 you must go and check-up for cancer.’ (Participant 19, 69 years, Pensioner)

‘I only got a pamphlet today … yea and I read it.’ (Participant 14, 78 years, Pensioner)

‘They showed me that book about prostate cancer. That lady “Mosa” she showed us that book about prostate, and that’s when I started to hear that I have prostate.’ (Participant 9, 72 years, Pensioner)

### Theme 3: Symptoms experienced during screening

Participants in the study reported that most of the time they experience pain and discomfort during the process of prostate cancer screening. In the current study, participants were asked about their experiences regarding the screening procedure at the specific tertiary care hospital. Two categories emerged, namely: (1) pain and (2) discomfort.

#### Category 3.1: Pain

The study revealed that the majority of the patients experienced pain and some discomfort during screening. The most severe pain reported was associated with the digital rectal exam procedure. They reported that the pain came when the health practitioner inserted an object in their rectum in search for prostate cancer. Their narratives were as follows:

‘Eish pains! it is very painful [*laughing – patient* {*embarrassed*!!}]. But he [*the doctor*] told us before the procedure that it is painful, but as this is the normal procedure you must tolerate the pain. And indeed! I did tolerate.’ (Participant 3, 84 years, Pensioner)

‘When they insert that thing [*probe*], it was difficult getting in and it was painful [*loudly emphasising this*!!]. Yea! it is very painful! And the time he was removing that thing then I was even able to breathe now.’ [*getting a sense of relief*]. (Participant 4, 71 years, Pensioner)

‘Yoh! it is very painful. Yea it is really very painful. But if you want to live you will just tolerate the pain so that you can live.’ (Participant 10, 62 years, Pensioner)

Further another participant described the pain followed by bleeding as follows:

‘Nah!! [*emphasising*], that one is the worse pain you can experience in your lifetime! I don’t think there is any other pain more than that, anything more than that then it is death. That was really close to death yeah!’ (Participant 17, 54 years, Unemployed)

Other participants further said:

‘It is not nice at all because it is like someone is shooting you straight at the prostate with a needle 12 times … you pass blood. Yes, it is not blood mixed with urine, you only pass blood.’ (Participant 15, 62 years, Pensioner)

‘I felt so dizzy! I felt dizzy!! [*emphasising*], and he said [*the doctor*] nah! you must sit. I felt dizzy and a lot of pain [*emphasising*!]. Then after 10 minutes then I felt the dizziness coming to an end, then he put me on a chair and said I should sit there for half an hour.’ (Participant 3, 84 years, Pensioner)

#### Category 3.2: Discomfort

Despite the fact that some reported pain, others saw the lighter side. Their narratives were as follows:

‘I don’t know the name of the instrument that was inserted at the back because they had inserted it at the back something that grasp a bit, you see. That is the only thing I felt but just a little bit.’ (Participant 5, 70 years, Pensioner)

‘Well, when they started, I felt uncomfortable. When they started to insert the finger to feel anything at the back of me because he wants to feel any irregularities you just feel uncomfortable. Yeah! because usually, there is nothing that enters there at the back yah that’s it.’ (Participant 15, 62 years, Pensioner)

‘I mean anything they stuck in your body it makes you uncomfortable.’ (Participant 12, 76 years, Pensioner)

‘I am going to speak the truth. I did not like it when he inserted a finger inside of me. But then I just accepted it because this person is a doctor. So, I must accept what he says we should do.’ (Participant 7, 54 years, Unemployed)

Despite the fact that some participants had negative experiences regarding the digital rectal exam, others had something positive to say. The participants expressed excitement knowing that they were going to receive help in this regard. The narratives were as follows:

‘Am no … What can I say [*seem unsure*!] I can’t mention how I felt. I was just happy that I am getting treatment and that I am going to be strong again [*referring to penis*].’ (Participant 4, 71 years, Pensioner)

‘Oh, yea it was not too bad. It was not too bad because I knew they are working yea [*implying its part of the process*].’ (Participant 13, 58 years, Unemployed)

‘Nah! because I want life, I did not have a problem with the process … yea I agreed, and I said they should check everything that they need to check.’ (Participant 8, 75 years, Pensioner)

‘I don’t have any problem, not at all. That’s why I am saying I am happy that I came here and found out that it is starting and go along with it, that’s it.’ (Participant 2, 57 years, Unemployed)

## Discussion

The purpose of the study was to explore the barriers and sources of information regarding prostate cancer screening at a specific hospital in Gauteng. The majority of older participants, particularly those aged 67 years, were more informed and proactive, and those residing in rural areas and those with lower income experienced significant barriers and had poor healthcare access. Cultural backgrounds and marital status affected decision-making especially among Zulu participants who viewed screening as a life saver. The findings of our study were disconfirmed by Mutua et al.^13^ in a study conducted in Kenya to investigate cultural factors associated with prostate cancer screening and they found no association with age, education, marital status and fear with regard to screening.

The present study found that media were the biggest source of information regarding prostate cancer, with none of the participants citing healthcare practitioners as their source of information about the disease. Also, few of the participants in the study reported that the sources of information were information pamphlets. In a study to assess information sources in patients with localised prostate cancer, Chhatre et al.^14^ reported that half of the participants identified the media as a source of information. Although this study did not identify healthcare providers as a source of information, participants in a study by Chhatre et al.^14^ identified healthcare providers as useful in communicating decision-making and treatments for prostate cancer. Another study by Gebru et al.^15^ recognised healthcare providers, media, family or friends and support groups as sources of information. These findings indicate that there is a gap in communication between health practitioners and patients regarding the provision of information at a specific academic hospital.

Barriers to screening in the study were fear, shyness, embarrassment and a lack of information pertaining to the disease. This finding from our study supports what was reported by Yeboa-Asiamah et al.^16^ in Ghana, who also found that fear, lack of information, and embarrassment were cited in their study.^9^ Similar findings were made by Ngowi et al.^17^ regarding prostate cancer knowledge and barriers to screening among men in communities of northern Tanzania. They found that the digital rectal exam procedure was described as invasion of privacy, harmful, embarrassing and that men feared a finger being inserted in the rectum. However, this finding contradicts that of Yeboah-Asiamah et al.^16^ in the Sunyai municipality in Ghana who found that the majority of participants disagreed that prostate cancer screening was embarrassing. The findings by Awosan et al.^16^ concur with those of the current study, which indicate that the lack of information was a barrier to screening.

Further, the study found that the symptoms experienced during the screening procedure were pain and discomfort as reported in other studies too.^18,19^ Elhardello and MacFie^20^ reported that most of the time patients experience pain and discomfort during the process of prostate cancer screening. However, Yeboah-Asiamah et al.^21^ in their study highlighted the fact that some of their participants disagreed with the fact that the screening for prostate cancer was painful. The present study also found some positives regarding the screening as some participants expressed excitement of receiving medical attention and relate the screening with cure. Similar findings were reported by Gellerstedt, Langius-Eklöf, Kelmendi, Sundberg and Craftman^22^ who found that the study participants regarded screening as a life saver.

The study has some limitations. Because of the qualitative nature of the study, the sample size was small and could not be generalised to all men attending the specific academic hospital. Socially desired responses might have been given by participants because of individual face-to-face in-depth interviews, or even possibly having discussed it with others as interviews were conducted on alternate days. Some participants might have shared some questions.

## Conclusion

The study provided evidence that a lack of privacy, pain control, fear of the procedure and embarrassment were barriers towards screening. The failure of healthcare practitioners to recommend screening when in contact with patients who are eligible also contributed as a barrier. Media and pamphlets played a role as sources of information regarding awareness pertaining to the disease.

The study findings are important as they highlight critical areas to improve the uptake of prostate screening. The identified screening barriers should be addressed through targeted strategies such as improving health information regarding screening for prostate cancer using culturally appropriate and respectful language. Improving communication between healthcare providers and patients and ensuring management of pain and provision of privacy are also required.

Future research should explore the effectiveness of intervention strategies aimed at addressing prostate screening barriers and investigating the role of sources of information in greater detail. Also, further research regarding communication on breaking the bad news on the prostate cancer diagnosis is required.

### Recommendations

Healthcare practitioners should be proactive in recommending screening to men who are eligible or might be at risk. It is important that healthcare practitioners are sensitive and exercise caution in maintaining privacy during screening. It is advisable that men are informed regarding what screening procedures entail to gain their cooperation and they should be provided with a light analgesic to ease discomfort during screening.
